# Understanding Volatile Electrical Switching in hBN Nanodevices by Fully Optical Operando Investigation

**DOI:** 10.1002/smll.202410569

**Published:** 2025-05-13

**Authors:** Dawn M. Kelly, Joanna Symonowicz, J. Callum Stewart, Stephan Hofmann, Giuliana Di Martino

**Affiliations:** ^1^ Department of Materials Science and Metallurgy University of Cambridge 27 Charles Babbage Rd Cambridge CB3 0FS UK; ^2^ Department of Engineering University of Cambridge Cambridge CB2 1PZ UK

**Keywords:** 2D materials, hexagonal Boron Nitride, in‐situ measurement, spectroscopy

## Abstract

Memristors based on 2D materials have emerged as promising candidates for use in artificial synaptic devices and energy‐efficient neuromorphic computing. Limited understanding of the fundamental switching mechanisms hinders device optimization and stalls commercialization. Conventional analysis techniques are often destructive, and only offer a static characterization of the device after electrical cycling, providing limited insights into switching dynamics. In this study, an operando approach utilizing plasmon enhancement of optical signals is used to investigate a two‐terminal vertical device based on monolayer hexagonal boron nitride. Real‐time photoluminescence and dark‐field scattering measurements reveal that conductive filaments (CFs) form through the migration of metallic ions from the electrode. The modification of a photoluminescence signal near 620 nm and a redshift of dark‐field scattering indicating a refractive index change of roughly 1 are detected when voltage is applied across the nanodevice. These optical changes are interpreted to show that this CF formation is mediated by point defect structures. This highlights the crucial role of defects in the switching dynamics. This finding resolves the ongoing debate in the literature about the mechanism of CF formation and paves the way for defect engineering as a step‐changing pathway to the optimization of these devices.

## Graphical abstract

Operando optical characterization of vertical two‐terminal monolayer hexagonal Boron Nitride devices is used to gain new insight into the key role of defects in the dynamics of switching. Characterization of photoluminescence and dark‐field scattering light signals during electrical switching indicates that the formation of conductive filaments of gold ions is mediated by vacancy defects.

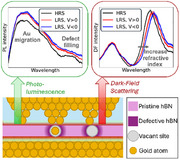

## 1 Introduction

In recent years there has been much interest in two‐dimensional (2D) materials as a potential device material for use in electronics.^[^ ^1^, ^2^, ^3^, ^4^ ^]^ In the context of ballooning demand for processing and storage of digital information, it is critical that devices not only continue to deliver more powerful computation capabilities but do so at a lower energy cost. The use of memristors for neuromorphic computing is one promising avenue: by allowing logic‐in‐memory computing architectures, memristors can overcome the Von Neumann bottleneck and thereby dramatically reduce energy consumption per calculation,^[^ ^5^ ^]^ in some cases by up to three orders of magnitude.^[^ ^6^ ^]^ In particular, hexagonal Boron Nitride (hBN) is a wide bandgap material that is considered highly suitable for electronics,^[^ ^7^, ^8^, ^9^, ^10^ ^]^ as a lack of dangling bonds or charge traps minimizes inelastic scattering of charge carriers.^[^ ^11^ ^]^ Other favorable qualities include hydrophobicity, high thermal conductivity, and the possibility to integrate into flexible electronics. When used for memristive devices, retention of 3 months has been demonstrated for a monolayer Au/ Cr/ hBN/ Au device,^[^ ^12^ ^]^ and endurance across 800 switching cycles has also been reported.^[^ ^13^ ^]^ It has been demonstrated that the use of lower or higher current limitation can lead to volatile or nonvolatile resistive switching respectively,^[^ ^14^, ^15^ ^]^ although in another study volatile threshold switching was observed at all current limits measured.^[^ ^16^ ^]^ In one case, nonvolatile bipolar switching was reported for a hBN device using gold for both electrodes, while volatile threshold switching was observed when silver was used for both electrodes.^[^ ^17^ ^]^ In general, the performance requirements for commercialization^[^ ^18^ ^]^ have not been fully met by any 2D memristive device. Variability and retention are two of the major barriers to commercialization, as illustrated by the review of hBN memristive devices given in **Table** **1**. Detailed studies on the mechanism of device switching are required to allow the sources of those limitations to be identified and mitigated.

**Table 1:** Summary of two‐terminal vertical hBN‐based memristors in literature, including devices that use a range of electrode materials and various hBN thicknesses. Conductive filaments are suggested to have been formed by metallic ions, boron vacancies, or by a mixed mechanism involving both.

Source	Device architecture	Thickness	Fabrication	Analysis method	Proposed mechanism	Retention
[12]	Au/ Cr/ hBN/ Au	1 layer	CVD^a)^ on Cu + wet transfer	I‐V	–	>90 days
[13]	Au, Cu or Ag/ PMMA^c)^ / hBN/ Cu	1 layer	CVD on Cu	I‐V, CAFM^b)^, ab‐initio sim.	Metal substituting V_B_ ^d)^ to form CF^e)^	>27h
[31]	Au/ hBN/ Au; Au/ hBN/ Ni	1 layer	CVD on Au or Ni + PMMA transfer	I‐V, ab initio sim.	Au substituting V_B_ to form CF	>7 days
[32]	Ag/ BNO_x_ ^f)^ / MLG^g)^ / SiO2	2–5 layers	Exfoliated, plasma oxidation, +dry transfer	Monte Carlo sim.	Ag CF	>4h
[33]	Au/ Ti/ hBN/ MLG/ MoS_2_	≥3 layers	Exfoliated + dry transfer	I‐V	Ti CF	–
[34]	Ag/ hBN/ Cu; Ag/ hBN/ ITO; ITO/ hBN/ ITO	3 nm	CVD on Cu	I‐V, CAFM, TEM^h)^	Ag CF	≈1h
[35]	Au/ Ti/ hBN/ Cu	>3 layers	CVD on Cu	I‐V	Ti or Cu CF	–
[36]	Au/ Ti / hBN/ Au	5–7 layers	CVD on CuNi^k)^	I‐V, EELS^l)^, CAFM, simulations	V_B_ CF	–
[36]	Au/ Ti/ MLG/ hBN/ MLG/ Cu	5–7 layers	CVD on CuNi, + transfer	I‐V, EELS, CAFM, simulations	V_B_ CF	≈11h
[37]	Au/ Ti/ MLG/ hBN/ MLG/ Au	5–7 layers	CVD on CuNi, + transfer	EELS, I‐V	CF from Ti and V_B_	≈11h
[37]	Au/ Ti/ h‐BN/ Au	5–7 layers	CVD on CuNi, + transfer	EELS, I‐V	CF from Ti and V_B_	–
[14]	Au/ Ti/ hBN/ Au; Pt/ h‐BN/ Cu	5–7 layers	CVD on Cu + PMMA transfer	I‐V, CAFM	Ti CF by B migration	–
[38]	Au/ Ti/ hBN/ Au	3 nm	CVD on Cu + PMMA transfer	I‐V	Ti CF by B migration	>24h
[39]	ITO /hBN /MLG/ PDMS^m)^	5 nm	CVD on Cu +PMMA transfer	I‐V, TEM, ED*S* ^n)^	In CF	≈14h
[16]	Al/ Ni/ hBN/ Ni	5nm	PMMA transfer	I‐V, TEM	Ni CF mediated by V_B_	–
[15]	Au/ Cr/ hBN/ Cu	10–12 layers	CVD on Cu	I‐V, TEM, CAFM	V_B_ CF	100s
[40]	Au/ hBN/ MLG/ hBN/ Ag	15 nm	Exfoliated + dry transfer	TEM, sims	Ag CF in one hBN layer & V_B_ CF in one hBN layer	≈11 days
[41]	Ag/ hBN+ PVOH^o)^/ ITO	Multi‐layer	liquid exfoliation + mix with PVOH	I‐V	Ag CF	≈3h
[17]	Au/ hBN/ Au; Ag/ hBN/ Ag	Multi‐layer	CVD on Cu+ PMMA transfer onto SiO2	TEM CAFM	–	–
[42]	Ag/ Al2O3/ GQD^i)^ + hBN / Ag/ PET^j)^	80 nm	GQD/hBN nanocomposite	I‐V	Ag CF	≈2 days

^a)^ CVD = chemical vapor deposition;

^b)^ CAFM = conductive atomic force microscopy;

^c)^ PMMA = polymethyl methacrylate;

^d)^ V_B_ = boron monovacancy;

^e)^ CF = conductive filament;

^f)^ BNO_x_ = amorphized boron oxide;

^g)^ MLG = multi‐layer graphene;

^h)^ TEM = transmission electron microscopy;

^i)^ GQD = graphene quantum dots;

^j)^ PET = polyethylene terephthalate;

^k)^ CuNi = Ni‐doped Cu;

^l)^ EELS = electron energy loss spectroscopy;

^m)^ PDMS = polydimethylsiloxane;

^n)^ EDS = energy‐dispersive spectroscopy;

^o)^ PVOH = polyvinyl alcohol.

Defects in hBN have been extensively studied, not only for their role in device switching and failure but also in the context of single photon emission at room temperature, which is of great interest in the field of quantum photonics.^[^ ^19^, ^20^ ^]^ Optically Detected Magnetic Resonance (ODMR)‐active defects are of particular interest, as these have potential for quantum‐photonic applications. High‐resolution transmission electron microscopy (HRTEM) validation has identified stable intrinsic defects in hBN, including monovacancies of Boron (V_B_)^[^ ^21^, ^22^, ^23^ ^]^ and Nitrogen (V_N_);^[^ ^21^, ^22^ ^]^ compound vacancies such as a pair of B and N atoms (V_BN_)^[^ ^21^ ^]^ the anti‐site nitrogen‐vacancy (N_B_V_N_);^[^ ^24^ ^]^ and tetra‐vacancies involving the absence of one N and the three surrounding B (V_B3N_),^[^ ^21^, ^22^ ^]^ or one B and the three surrounding N (V_B1N3_).^[^ ^25^ ^]^ Substitutional defects involving Carbon and Oxygen have also been observed by TEM.^[^ ^26^ ^]^ V_B_ are reported as the most common point defect in hBN monolayers grown by chemical vapor deposition (CVD),^[^ ^21^, ^23^, ^27^ ^]^ with one TEM study highlighting that the majority of boron vacancies observed had carbon in at least one of the nitrogen sites along the vacancy edge (N_C_V_B_).^[^ ^28^ ^]^ Charged V_B_^‐^ monovacancies have been investigated for use in optical spin readout schemes, with photoemission near 850 nm.^[^ ^29^, ^30^ ^]^ Characterisation of such defects at the atomic level remains very challenging, in particular when considering dynamic behavior and reaction kinetics.

hBN‐based two‐terminal vertical memristors in literature vary widely according to the thickness of hBN used and the materials selected for use as top electrode (TE) and bottom electrode (BE), with the most commonly proposed switching mechanism being the formation and rupture of metallic filaments, corresponding to low resistance state (LRS) and high resistance state (HRS) respectively (Table 1). Conductive filaments (CFs) composed of boron vacancies (V_B_) are thought to form in devices with inert electrodes such as graphene,^[^ ^36^, ^40^ ^]^ although in ref. [36]. A V_B_ CF was postulated for both a device with Ti TE and Au BE and one with the hBN enclosed by multilayer graphene (MLG) on both sides: Au/ Ti/ MLG/hBN/MLG/Au. In ref. [40] a tri‐layer hBN/MLG/hBN structure was contacted using Au TE and Ag BE, and the authors concluded that CFs formed from boron vacancies in the top hBN layer and from Ag ions in the bottom layer. However, not all studies using inert electrodes yielded the same results: ref. [34] saw no switching in an ITO/hBN/ITO device, while ref. [39] shows that ITO provides metal ions for CF formation in an ITO/ hBN/ MLG/ PDMS device. A recent ab initio study on switching in hBN devices with inert electrodes suggested conduction via the clustering of monovacancy defects.^[^ ^43^ ^]^

Some experimental studies discuss the mechanism of metallic CF formation, postulating the involvement of boron vacancies and highlighting the increased density of boron vacancies along grain boundaries,^[^ ^13^, ^14^, ^16^, ^31^, ^37^, ^38^ ^]^ with Electron Energy Loss Spectroscopy (EELS) finding migration of metal ions into the hBN layer^[^ ^16^, ^34^, ^36^, ^37^, ^39^, ^40^ ^]^ as well as migration of boron toward the anode in devices after they have been cycled.^[^ ^36^, ^37^ ^]^ The involvement of grain boundaries is evidenced by some authors who note that exfoliated flakes, which will generally have higher crystal quality and fewer grain boundaries, do not exhibit any resistive switching behavior.^[^ ^37^, ^38^, ^44^ ^]^ Conversely, others report an observation of resistive switching in devices using mechanically exfoliated hBN.^[^ ^33^, ^40^ ^]^ A key challenge to the field remains the direct characterization of the switching to fingerprint the key underpinning mechanisms and rationalize the diverse findings across the literature. This motivates us here to utilize a Nanoparticle‐on‐Mirror (NPoM) geometry^[^ ^45^, ^46^, ^47^, ^48^, ^49^, ^50^ ^]^ to enable a nano‐optical operando investigation of memristive switching in such vertical hBN device stacks. The use of the plasmonic NPoM geometry allows the collection of optical information from a nano‐volume of the material, overcoming the diffraction limit that other optical tools are constrained by. The plasmonic enhancement of optical emissions by the NPoM allows measurements that are highly sensitive to local changes within the switching material, resulting in a powerful tool for rapid nanoscale device characterization. In this work, the NPoM platform is used to examine photoluminescence and dark‐field scattering signals from the hBN monolayer. Environmental factors such as humidity have been shown to have a strong impact on the performance of hBN‐based devices.^[^ ^51^, ^52^ ^]^ The NPoM geometry allows for all measurements to be under ambient conditions, maximizing the relevance of the results to the real‐world applications of the studied materials.

## 2 Experimental Results

**Figure 1:**
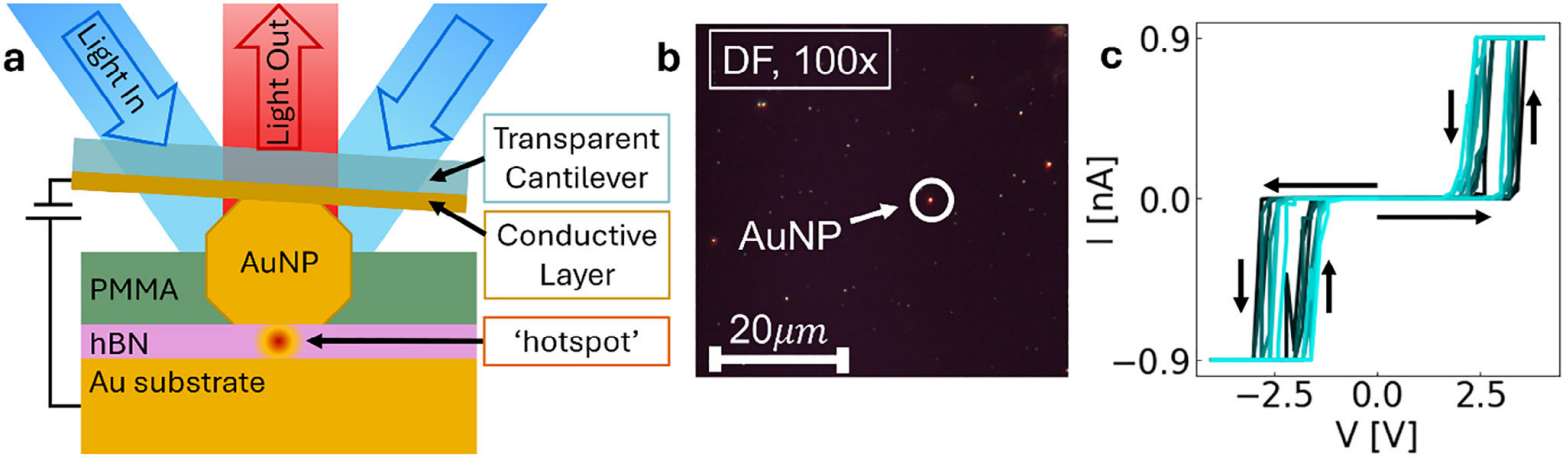
In situ optical investigation of hBN‐based memristive device. a) Schematic of cantilever setup allowing simultaneous optical and electrical characterization. b) DF optical microscopy image of AuNP on hBN. An AuNP, which would later be electrically contacted using the cantilever, which is 35 µm in width, is highlighted. c) *I–V* curves showing threshold resistive switching of an hBN device. The current compliance was set to 0.9nA.

A nanoswitch based on monolayer hBN was constructed in a NPoM, as illustrated in **Figure** **1**a. The monolayer is hosted on an Au substrate, which acts as the bottom electrode for the device. While the deposition of conventional evaporated Au electrodes often requires an additional interfacial Cr or Ni adhesion layer, here an 80nm‐diameter gold nanoparticle (AuNP) is deposited directly onto the test material by drop‐casting. The AuNP is estimated to have a facet diameter of 30 nm, giving a device cross‐section of *A* ≈ 700 nm^2^. The AuNP is held in place only by Van der Waal forces, allowing for the direct contact of the AuNP top electrode with the hBN monolayer. An optically transparent quartz‐like cantilever is used to electrically contact the AuNP, allowing for the hBN device to be electrically switched while optical signals are collected simultaneously. White light illumination excites plasmons in the AuNP which interact via mirror charges with the gold substrate below to form a coupled plasmonic system, with a “hotspot” of enhanced field intensity focused on the test material between the two metallic regions. Dark Field (DF) spectroscopy measures the frequency change, i.e., color change, of this coupled plasmonic response and is highly sensitive to any material changes happening in the space between the gold substrate and NP, e.g. the refractive index of the material in the gap. Figure 1b shows a DF optical microscopy image of the hBN sample. The measured *I–V* curves (Figure 1c) illustrate the device’s volatile threshold switching. Under positive applied bias, the device reaches LRS at a mean voltage of 3.1 V and returns to HRS when voltage drops below 2.5 V; while for negative applied bias, the device reaches LRS at −2.6 V and reverts to HRS at a mean value of −1.8 V. The asymmetry of the threshold voltages may be due to the asymmetrical electrodes utilized in the device. The larger magnitude of the SET voltage in the V > 0 case can be attributed in part to the presence of the stabilizing ligands on the surface of the AuNP, since under positive applied voltage the AuNP acts as the source of Au for the formation of a CF. The presence of ligands does not alter the overall switching behavior. Application of voltage > 4 V led to irreversible dielectric breakdown, followed by the destruction of AuNP electrodes. This breakdown is identified by discrete and drastic change in the DF signal (Figure S1, Supporting Information). The final breakdown of the device discussed below occurred after ≈400 switching cycles.

### 2.1 Operando Photoluminescence Measurements

A 444 nm laser is used to illuminate the device. Our geometry allows the excitation and collection of Photoluminescence (PL) signals from the nanovolume within the plasmonic hotspot generated by the NPoM, hence enabling the tracking of defect signatures within the hBN nanodevice. While other techniques, such as chemical analysis in TEM, one may only study a vertical device “postmortem,” this approach offers an opportunity to resolve switching dynamics in real time.

**Figure 2:**
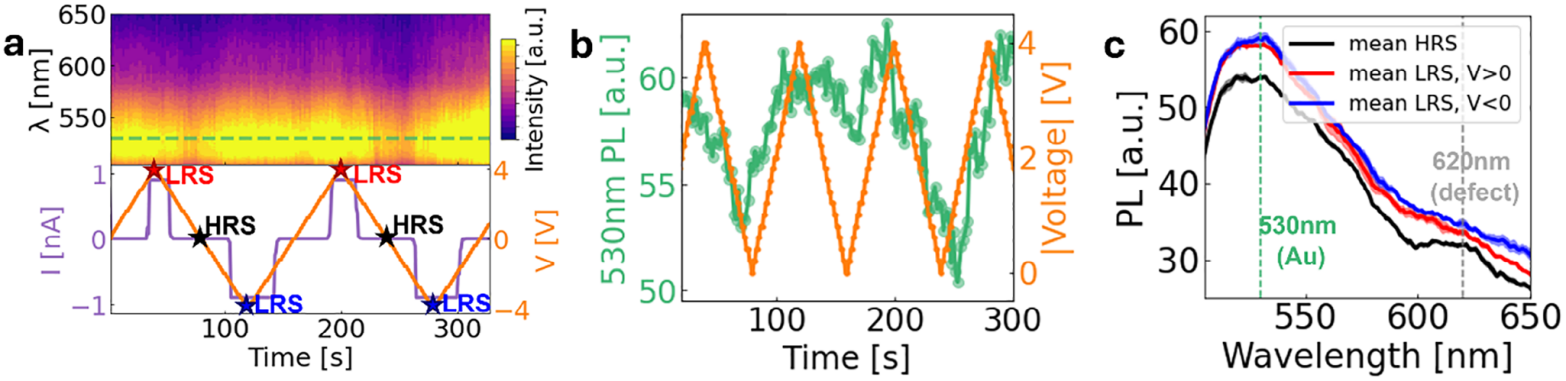
Operando photoluminescence measurements. a) Simultaneous IV and PL characterization (current limitation was 0.9nA); b) 530 nm peak intensity variation over three switching cycles showing voltage dependence; c) Mean PL signals for HRS and LRS of the hBN nanoswitch with shaded areas representing error due to averaging.

We track the PL signal against time, with the voltage applied across the device and the resultant current flow plotted against the same time axis (**Figure** **2**a). A scaling factor has been applied to the PL spectra to account for stage drift causing a gradual decrease of overall signal intensity over time, as is illustrated in Figure S2 (Supporting Information). A strong PL signal, consisting of a broad peak centered near 530 nm (Figure 2a, green dashed line), is visible and commonly attributed to gold.^[^ ^53^, ^54^ ^]^ The 530 nm peak intensity variation over a few switching cycles is compared with the magnitude of the applied voltage (Figure 2b), illustrating that both quantities follow a similar pattern of variation over time. This indicates the reversible voltage‐drive motion of Au atoms from the electrodes into (LRS) and out (HRS) of the plasmonic hotspot, which sits in the hBN monolayer. The Au‐driven switching behavior resembles that of another 2D resistive system, as exemplified by our recent study on MoS_2_.^[^ ^55^ ^]^

Furthermore, a closer look at the PL signal in the HRS (Figure 2c, black) reveals an additional peak at ≈620 nm region, which is assumed to originate from a point defect in the hBN monolayer. Independent of the identity of the defect, we note that its emission is absent in the LRS‐type spectra (Figure 2c, red and blue). This implies that upon switching from HRS to LRS the defect is modified such that it no longer emits at this wavelength, possibly due to the introduction of an Au atom. In the absence of defects, the hBN monolayer is impermeable to anything larger than a proton,^[^ ^56^, ^57^ ^]^ so we may assume that any defect which allows the introduction of an Au atom to the hBN monolayer will include a lattice vacancy. Significantly, when biased exfoliated (defect‐free) flakes were tested using the cantilever setup described above they did not exhibit any memristive loops. This further supports the conclusion that defects are involved in the memristive switching of hBN monolayers.

The observed variation in PL may then be explained as follows: the applied voltage drives the migration of gold atoms from the electrodes into defects which are assumed to include vacant sites within the hBN, forming conductive filaments between electrodes such that the device switches from HRS to LRS. The structure of the vacancy defect is modified by the introduction of the Au such that it no longer emits at 620 nm when the device is in LRS.

### 2.2 Operando Dark Field Spectroscopy

**Figure 3:**
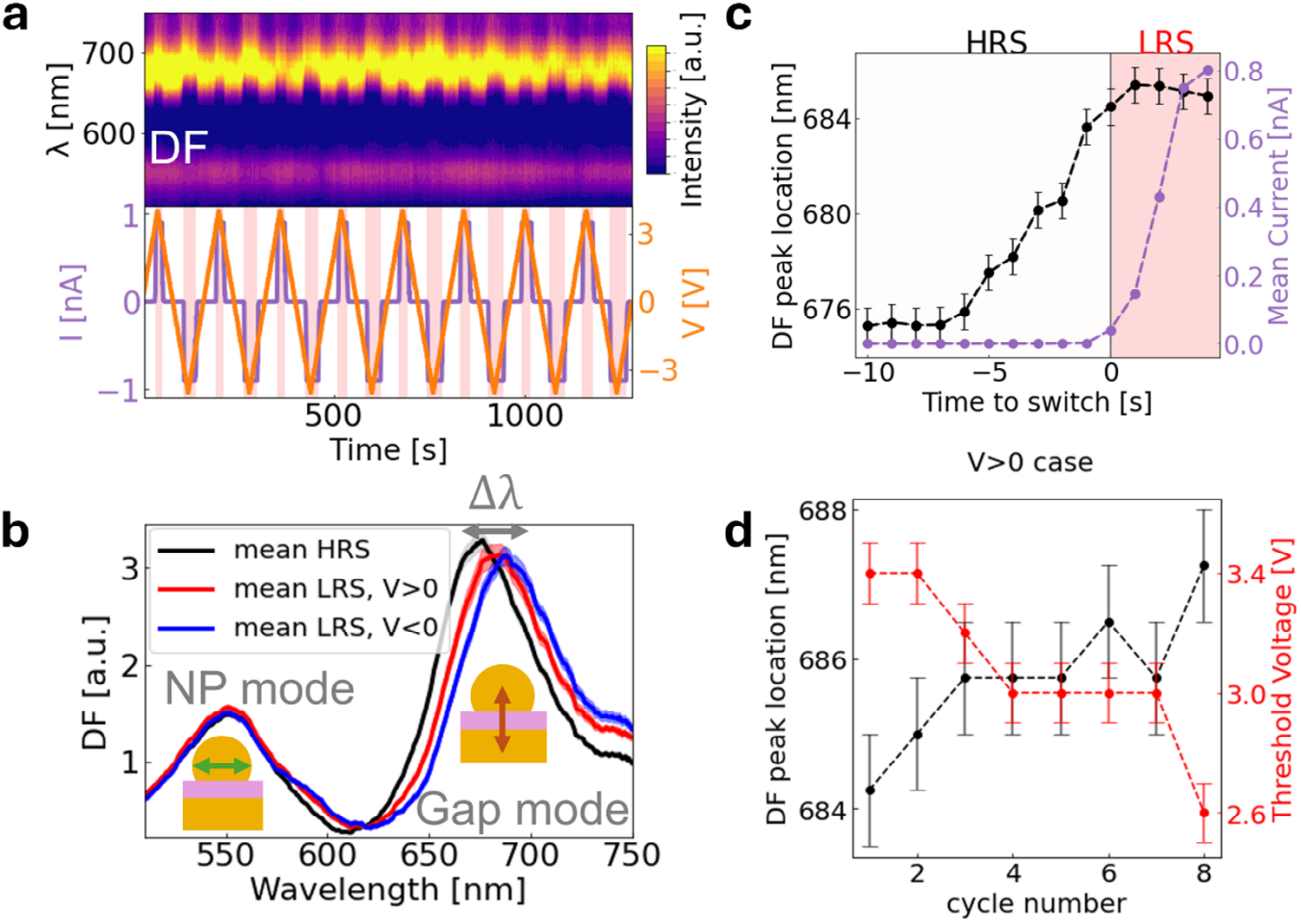
Operando DF scattering measurements a) Simultaneous IV and DF characterization b) Mean DF signal, calculated when programmed voltage was 0, +4, and −4 V are shown in black, red, and blue respectively. A redshift of the gap mode peak is evident when the voltage is high. c) The average location of the DF gap mode peak relative to the time of current onset, for all switching events under positive voltage. d) The DF peak location 4 s after current onset for each switching event under positive voltage shows an increasing trend when plotted against cycle number, while the voltage required to switch from HRS to LRS shows a decreasing trend.

To corroborate this result, we study DF signals in situ upon application of triangular bias programmed to range from 4 to −4 V, with current compliance set to 0.9nA (**Figure** **3**a). The peak at ≈550 nm represents the plasmon hosted in the “AuNP single mode” (Figure 3b, green arrow), which is not affected by the switching and hence stays in a fixed spectral location during switching, as expected. The peak at ≈685 nm is the scattering from the coupled plasmon mode or “gap mode” plasmon (Figure 3b, orange arrow). Similarly to the processing applied to the operando PL data, a scaling factor has been applied to the in situ DF spectra to correct for overall signal intensity falling over time (Figure S2, Supporting Information). The spectra taken at 0 V (device in HRS – Figure 3b, black), show mean gap mode at 675 nm, while the mean peak locations for spectra measured at a maximal positive or negative voltage (device in LRS – Figure 3b, red and blue) are redshifted to 684 and 689 nm, respectively. We note that the redshift of the gap mode peak is greater for negative voltages when the Au substrate acts as the anode and the nanosized AuNP acts as a cathode, i.e., Au ions migrating from bottom surface to top NP contact.

The switching events under positive applied voltage (V > 0) were isolated, and the mean DF spectrum relative to current onset was calculated (Figure 3c). The mean initial peak location is 675 nm, and the peak shift begins at a mean value of 2.1 V. LRS is achieved at a mean voltage of ≈3.1 V, with a mean peak location of 684 nm. It is evident that the redshift of the gap mode peak is essentially completed by the time of current onset (LRS), with the mean peak location only increasing by 1 nm further after the current onset (Figure 3c). It is relevant to note that the magnitude of applied voltage required to switch the device from HRS to LRS decreased over time during the operando DF measurement, (Figure 3d), and simultaneously the redshift became more pronounced. These trends imply that the morphological change within the hBN causing the redshift is a condition necessary to enable current to flow, and that this change became more pronounced as the device was cycled.

## 3 Discussion

Our operando PL data features a peak near 620 nm which is observed only in the HRS. The following discussion briefly reviews photoemission from hBN in this spectral region, for the purpose of confirming that there are point defect structures in hBN that may explain this emission. Multiple works have postulated the involvement of carbon in hBN defects with photoemission near 620nm: for example, a density functional theory (DFT) study of a range of defects including carbon, oxygen, and silicon substitutions assigned V_N_C_B_ as the most likely source of emission near 635 nm.^[^ ^58^ ^]^ An examination of a neutral V_N_C_B_ defect using group theory and DFT concluded that it would be stable mainly in *n*‐type BN monolayers, and calculated the photoemission wavelength as close to 595 nm.^[^ ^59^ ^]^ Another DFT study also highlighted V_N_C_B_ as the strongest candidate for emission near 635 nm, due to this defect giving the best agreement between experimental and theoretical Huang–Rhys factor and emission lineshape out of those considered.^[^ ^60^ ^]^ Elsewhere, the V_N_C_B_^‐^ defect has also been presented as a likely candidate for the origin of visible PL observed in hBN.^[^ ^61^ ^]^ Given the organic long‐chain surfactants used to stabilize the AuNPs used in this work, carbon atoms would be readily available at the interface between the hBN monolayer and the AuNP electrode and so defects involving carbon substitutions should certainly be considered among candidates. The anti‐site complex N_B_V_N_ has also been proposed as a possible origin of emission near 620 nm, justified by a combination of group theory analysis and DFT.^[^ ^62^ ^]^ Experimental observation of both the energy and anisotropy of emission near 625 nm from monolayer hBN has also been matched by DFT predictions of the N_B_V_N_ defect.^[^ ^24^ ^]^ The authors of a recently published database of hBN defects calculated using DFT emphasize that multiple photophysical properties should be used simultaneously for the assignment of defects, rather than attempting to identify the source of any given emission using a single property.^[^ ^63^ ^]^ This need for multiple data points highlights the difficulty of defect identification in hBN. Local environmental factors, such as grain boundaries, must also be considered as they may alter the emission wavelength of defects in monolayer hBN, particularly given that the optical signals under consideration are collected from a small area (facet area of AuNP contact is expected to be 20–30 nm, where total AuNP diameter is 80 nm). For example, it has been shown that the presence of a wrinkle can change the distribution of defect emission wavelengths when compared to flat film.^[^ ^64^ ^]^ While PL emission of hBN has been demonstrated to be pressure‐dependent,^[^ ^65^ ^]^ it is not expected that sufficient pressure would be achieved in this system to have any effect on PL wavelengths.^[^ ^46^ ^]^ Theoretical analysis has demonstrated that fluorescence in hBN can also be strongly affected by strain.^[^ ^66^ ^]^ Clearly, there are many candidate defect structures for the emission we find at 620 nm and the specification of the identity of the defect responsible for this emission is beyond the scope of this paper.

The operando PL data also shows that the applied voltage drives the migration of gold atoms from the electrodes into defects within the hBN. Combining this with insights gained from operando DF, we want to assess the possibility of the mechanism of metal migration across the layer being facilitated not only by pre‐existing vacancy defects, but also that the applied voltage may cause the formation of new defects in the hBN such that Au may occupy the newly vacant site. This concept that the switching region becomes more and more defective is supported by the decrease of threshold switching voltage observed with ongoing device cycling (Figure 3d). This fact may have significant implications for device lifetimes. We note also that the transition from low to high current flow is not sharp, but shows fluctuations (illustrated in Figure S3, Supporting Information), which plots the current profile under a similar but more slowly varying triangular applied voltage profile). This “piecewise” transition from HRS to LRS is consistent with the formation of many atomic bridges across the hBN.

The operando DF data presented in Section 2.3 illustrated a consistent redshift of the spectral location of the gap mode plasmon upon switching from HRS to LRS. A redshift of the gap mode plasmon peak can in general be caused by *i*) decreasing of thickness of the material in the gap or *ii*) increasing the refractive index of the material in the gap. While it has previously been established that higher voltages lead to increased cantilever pressure on the test material, this has been estimated to be ≈ 0.13 GPa in our system.^[^ ^46^ ^]^ Such pressure is not expected to cause appreciable thickness change in the test material.^[^ ^55^, ^67^ ^]^ Our previous operando DF study of MoS_2_ nanodevices with identical NPoM geometry showed a decrease in the gap mode intensity upon switching from HRS to LRS, which we rationalized as being due to the formation of many thin nanofilaments (a conclusion supported by Finite‐Difference Time‐Domain (FDTD) simulations).^[^ ^55^ ^]^ These simulations also showed that the formation of thicker metallic filaments in the switching area would lead to a blueshift of the gap mode, rather than a redshift as is observed here. Hence this redshift of the DF gap mode peak in Figure 3b may consistently only be explained as due to increasing refractive index rather than any thickness decrease or due to the migration of Au.

**Figure 4:**
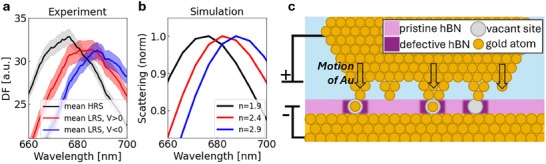
Finite‐difference time‐domain simulations are used to model material changes before and after switching. a) Mean DF signal gap mode peaks, calculated at HRS (zero applied voltage) and LRS (maximal positive and negative applied voltages) are shown in black, red, and blue respectively. b) Simulated scattering peaks for various refractive indices c) Schematic of proposed switching mechanism involving metal migration mediated by vacancy defects.

We therefore presentFDTD simulations^[^ ^68^ ^]^ used to model the hBN refractive index change that would be required to generate the change in the observed DF spectrum (**Figure** **4**). The baseline refractive index of hBN was taken as 1.9,^[^ ^69^, ^70^ ^]^ and an increase of + 0.5 (leading to n = 2.4) and +1 (leading to n = 2.9) were used to match the redshift of the mean DF spectrum measured at maximal positive and negative voltages, respectively. The greater redshift of the gap mode peak for negative voltages may be due to the larger surface area of the electrode from which Au ions migrate (in the V < 0 case the Au substrate acts source of Au ions, while in the V > 0 case it is the AuNP). Given that the device does not switch from HRS to LRS until the redshift of the DF peak is complete, we may infer that the voltage‐induced material change that allows current to flow is the same one causing this large increase in refractive index. This material change became more pronounced over time, with the magnitude of the redshift increasing with cycle number (Figure 3d). We now focus on the cause of this increase in local refractive index.

Evidence from multilayer vertical hBN devices emphasizes the importance of boron to the switching mechanism, with previous analysis of the cross‐section of devices after cycling using EELS having established significant migration of boron toward the anode.^[^ ^14^, ^71^ ^]^ Extrapolating this evidence to the monolayer system here, we consider defects that involve a vacancy, the formation of which would involve the displacement of at least one boron atom. Defect candidates for emission in this wavelength range that meet these criteria include V_N_C_B_,^[^ ^61^ ^]^ and N_B_V_N_.^[^ ^62^ ^]^ Given that hBN is a dense monolayer, these boron atoms would presumably be displaced from the monolayer plane. Working in the approximation that a region of displaced boron atoms would accumulate in the switching region while the device is in LRS, and considering that in the bulk boron has a refractive index in the range of 3–3.35 across the visible range,^[^ ^72^ ^]^ we note that the formation of a boron rich region in the plasmonic hotspot could be expected to increase the effective refractive index. Accumulation of displaced boron is therefore one possible explanation for the redshift of the DF gap mode peak in Figures 3b and 4a. The overall voltage response of the hBN device is then understood to be the following: the HRS to LRS transition under applied voltage occurs by driving the Au atoms to migrate from the electrode into (boron‐vacancy) defect sites within the hBN monolayer, forming a CF. The operando DF data evidence that the applied voltage may also drive some of the native boron in the hBN to overcome the energy barrier to displacement, forming a new vacancy defect structure which is then occupied by the Au which forms a part of the CF. The stability of the CF is sufficiently low that when the voltage returns to zero, CF breakdown occurs, leading to a volatile electrical response. This switch from LRS to HRS occurs as the Au migrates back to the electrode, allowing for the vacancy defect and the displaced boron to recombine, returning to a lower energy crystallographic configuration.

Therefore, our operando characterization not only corroborates the mechanism of metal ions moving to occupy defect sites in the hBN, but also further details the switching dynamics, adding the displacement of atoms to the picture. Our previous work offers a direct comparison to switching dynamics in MoS_2_, in which conductive filaments were also concluded to be formed by the migration of Au ions from the electrodes.^[^ ^55^ ^]^ However, unlike hBN, the in‐plane arrangement of atoms of MoS_2_ allows sufficient space for direct intercalation of Au. In fact, a vacancy‐assisted mechanism for MoS_2_ was ruled out, since the PL associated with sulfur vacancies (V_S_) remained unchanged during electronic switching of the studied devices.^[^ ^55^ ^]^ Given that switching did not involve any morphological change to the native material, the only avenue available for control of the ionic migration across the MoS_2_ device is surface engineering. For hBN, the electron orbitals do not leave any pores of sufficient size for any metal ion to pass through, necessitating the involvement of defects for any conductive filament to form. This affords the opportunity for device optimization via defect engineering in the hBN. In general, it may be concluded that the combination of the ionic radius of the electrode metal and the spatial distribution of the electron orbitals of a given 2D material provides a strong indicator as to which engineering routes are available for the modification of device performance.

## 4 Conclusion

The non‐destructive, correlative operando characterization technique used in this work has allowed direct insight into the switching dynamics within a hBN monolayer‐based memristive device stack. The combination of operando PL and DF measurements, allows us to conclude that the conductive path formation for the LRS is linked to hBN defect‐mediated Au migration. Our assignments indicate that such a mechanism involves B displacement. This offers clarification to the debate in the literature about the mechanism of CF formation in hBN devices, confirming the necessity of point defects. We also confirm defect creation during switching and highlight the possible impact of this on the device lifetime. Hence for these types of devices, defect engineering in hBN is a critical requirement for device optimisation. This holds for 2D materials like hBN where the “perfect” monolayer is highly inert and impenetrable, i.e., an effective diffusion barrier, to metal ions. Combined with our previous operando data, our results highlight that this is distinct to layered TMD materials like MoS_2_ with a more complex monolayer structure and lower lattice stability.

## 5 Experimental Section

### hBN Monolayer Growth

A custom cold‐wall reactor was used for the CVD growth of monolayer hBN.  As‐received polycrystalline platinum foil (25 µm, 99.99%, Alfa Aesar) was used to catalyze the reaction; supported by a tantalum susceptor (25 µm, 99.9%, Goodfellow), which was clamped to a ceramic mount using sapphire. The platinum was wrapped around the tantalum, away from this clamped region. A continuous wave 808 nm laser with a top hat beam profile of dimensions 5 × 5mm^2^ was used for localized heating of the sample. IR pyrometer was used to measure temperature, with a wavelength of 1.6 µm and spot size of 3 mm, and the emissivity set to 0.25. A base pressure of <2 × 10^−6^ mbar was used both for vacuum annealing and growth, and a temperature of ≈1090 °C was used. A borazine precursor (>97% Fluorochem) was dosed after 5 min vacuum annealing, at a partial pressure of 1.3 × 10^−5^ mbar, which was sufficiently low to avoid the occurrence of multilayer islands. Growth was allowed to continue for 25 min, which ensured complete coverage of the Pt catalyst with a continuous monolayer of hBN.

### hBN Monolayer Transfer

Cleaning of Au substrate was achieved by 15 min of sonication in a 40 °C acetone bath, followed by a further 15 min of sonication in isopropyl alcohol. The substrate was dried with an N_2_ gun after each sonication step. The Au substrate was annealed on a hot plate set to 135 °C for 10 min in air immediately before hBN transfer. A PVA solution formed of 5 g of PVA (Mw 9000–10000, 80% hydrolyzed, Sigma–Aldrich) and 1 g of glycerol (>99% Sigma–Aldrich) in 100 mL of deionized (DI) water was drop‐cast onto the hBN on the Pt catalyst. This was left on a hot plate at 60 °C for approximately an hour/until the PVA was dry.

The PVA layer then served as a backing support for the peeling transfer of the monolayer hBN from the catalyst to the Au substrate: the PVA/hBN was peeled away from the Pt catalyst and was placed onto a polydimethylsiloxane stamp. With the Au substrate on a hot plate at 135 °C, a rolling motion was used to stamp the hBN/PVA onto the Au. The stack was left on the hot plate for at least 5 min. Dissolution of the PVA after transfer was achieved by placing the stack in a DI water bath at 90 °C for 1 h.

### Electrical Characterization

A Keithley 2634B sourcemeter was used to carry out electrical measurements, connected to the sample electrodes via triaxial cables. The bottom electrode (Au substrate layer) was contacted using a tungsten probe tip from *Lambda Photometrics Ltd*, while the top electrode (AuNP, facet diameter ≈ 30 nm, contact area with film≈700 nm2) was contacted using tipless semi‐transparent cantilever (*Apex Probes*) of dimension 30 µm × 100 µm, coated using thermal evaporation with 3 nmCr/6 nm Au.  A custom mount using piezo‐controlled actuators (*from SmarAct*) was used to bring the cantilever parallel to the sample surface. The AuNP density was sufficiently low to allow a single AuNP to be contacted at a time, All measurements were performed under ambient conditions.

### Optical Characterization

All optical spectra were collected using a 100x, 0.8‐NA objective from *Olympus* (LPMlanFLN) integrated into a custom optical rig. A continuous wave 444 nm single‐longitudinal‐mode laser (*Integrated Optics)* was used for PL measurements, with laser power of ≈1.5 µW incident on the sample. White light illumination was used for DF scattering measurements. Both PL and DF signals were directed to an Oceans Optics QE65000 spectrometer using an optical fiber (model QP50‐2‐UV–vis).

### FDTD Simulations

Commercial *Lumerical* software from *Ansys* ^[^ ^68^ ^]^ was used to simulate light scattering response. The AuNP was modeled as a sphere of 80 nm diameter, with an interface with the material below formed by a cross‐section of the sphere with a diameter of 30 nm. A layer of 0.5 nm of ligands was modeled to be on the surface of the AuNP. The optical constants for gold were taken from ref. [73]. The refractive index of the ligand and residue layers used in the model was set to n = 1.5, based on the values found in the literature for PMMA,^[^ ^74^ ^]^ citrate,^[^ ^75^ ^]^ and polystyrene.^[^ ^76^ ^]^

## Conflict of Interest

The authors declare no conflict of interest.

## Supporting information



Supporting Information

## Data Availability

The data that support the findings of this study are openly available in Apollo at https://doi.org/10.17863/CAM.113049, reference number 1654185.
