# Supplementary material for: Understanding Volatile Electrical Switching in hBN Nanodevices by Fully Optical Operando Investigation
Source: Small. 2025 May 13;21(26):2410569. doi: 10.1002/smll.202410569 (PMC12232225; doi:10.1002/smll.202410569)
Supplement: Supplementary file 1 — Supporting Information [file SMLL-21-2410569-s001.docx]

**Supporting Information**

**Understanding Volatile Electrical Switching in hBN Nanodevices by Fully Optical Operando Investigation**

Dawn M. Kelly, Joanna Symonowicz, J. Callum Stewart, Stephan Hofmann, Giuliana Di Martino*


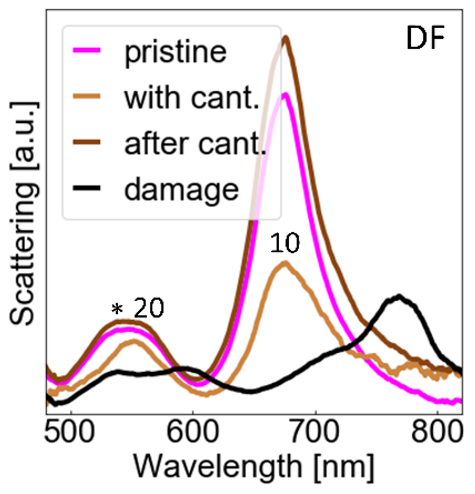


**Figure S1**. Dark-Field (DF) spectroscopy of monolayer hBN on Au substrate, showing that DF collected from a nanoparticle on hBN is unchanged by the use of cantilever. The spectrum labelled as ‘damage’ illustrates DF collected after device breakdown.


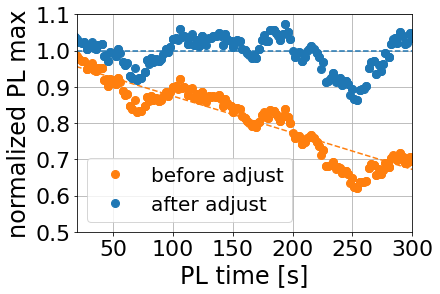


**Figure S2**. Illustration of data processing applied to account for stage drift. The maxima of the photoluminescence (PL) spectra are plotted versus time of acquisition and fitted to a straight line. A multiplication factor is applied to all spectra such that this straight line is horizontal. An analogous process flow was applied to the DF spectra, with the intensity of the NP single mode peak used as the quantity to be scaled rather than an absolute maximum as was used for the PL spectra.


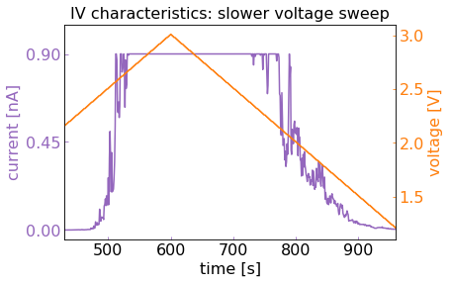


**Figure S3** A further electrical test in which a much more slowly varying triangular bias pattern was applied is plotted. The current plateau is due to reaching the current compliance setting. The transition from HRS to LRS is not sharp, but composed of many small steps, confirming that many conductive filaments are formed in the switching region.
